# Supplementary material for: Regenerative neurogenic response from glia requires insulin-driven neuron-glia communication
Source: eLife. 2021 Feb 2;10:e58756. doi: 10.7554/eLife.58756 (PMC7880684; doi:10.7554/eLife.58756)
Supplement: Transparent reporting form [file elife-58756-transrepform.docx]

***eLife’s* transparent reporting form**

We encourage authors to provide detailed information *within their submission* to facilitate the interpretation and replication of experiments. Authors can upload supporting documentation to indicate the use of appropriate reporting guidelines for health-related research (see [EQUATOR Network](http://www.equator-network.org/%20)), life science research (see the [BioSharing Information Resource](https://biosharing.org/)), or the [ARRIVE guidelines](http://www.plosbiology.org/article/info:doi/10.1371/journal.pbio.1000412) for reporting work involving animal research. Where applicable, authors should refer to any relevant reporting standards documents in this form.

If you have any questions, please consult our Journal Policies and/or contact us: [editorial@elifesciences.org](mailto:editorial@elifesciences.org).

**Sample-size estimation**

- You should state whether an appropriate sample size was computed when the study was being designed
- You should state the statistical method of sample size computation and any required assumptions
- If no explicit power analysis was used, you should describe how you decided what sample (replicate) size (number) to use

Please outline where this information can be found within the submission (e.g., sections or figure legends), or explain why this information doesn’t apply to your submission:

Sample sizes for all the experiments are provided in Supplementary File 1.

For antibody stainings in Drosophila larval CNSs, estimation of sample size was based on our previous experience, and we aimed as much as possible to analyse at least n=10 specimens from successful experiments. For some quantifications, we aimed for n≥15.

For injury experiments, many injured samples did not maintain sufficient integrity to reach the final stages of the procedure, and approximately five times more samples were injured than made it to the final steps.

For qRT-PCR, we aimed for 3 biological replicates, from n≥10 dissected larval CNSs each; each biological replicate was from a separate cross involving different groups of G0 females and males.

The statistical analysis is described in the Methods section and Supplementary File 1.

**Replicates**

- You should report how often each experiment was performed
- You should include a definition of biological versus technical replication
- The data obtained should be provided and sufficient information should be provided to indicate the number of independent biological and/or technical replicates
- If you encountered any outliers, you should describe how these were handled
- Criteria for exclusion/inclusion of data should be clearly stated
- High-throughput sequence data should be uploaded before submission, with a private link for reviewers provided (these are available from both GEO and ArrayExpress)

Please outline where this information can be found within the submission (e.g., sections or figure legends), or explain why this information doesn’t apply to your submission:

For immunostaining data, experiments were repeated as often as necessary until it was clear the stainings had worked. For successful stainings, only inappropriately mounted samples were excluded from the analysis; no outliers were excluded. Data were validated with reasonably large sample sizes, as indicated in section above.

For injury experiments, a large number of specimens was first injured, but some were lost in the process through excessive damage and subsequent handling of the injured CNS (dissection, fixation, staining, washing, etc. as these samples are fragile and can break apart). As a result, sample sizes for injury experiments ended up being smaller than for other experiments. No outliers were excluded.

For quantitative real time PCR data, between 2 and 4 biological replicates were used. For each replicate, n≥10 dissected larval CNSs were used; each biological replicate was from a separate cross involving different groups of females and males at G0.

All sample sizes, genotypes and repeats are provided in Supplementary File 1.

**Statistical reporting**

- Statistical analysis methods should be described and justified
- Raw data should be presented in figures whenever informative to do so (typically when N per group is less than 10)
- For each experiment, you should identify the statistical tests used, exact values of N, definitions of center, methods of multiple test correction, and dispersion and precision measures (e.g., mean, median, SD, SEM, confidence intervals; and, for the major substantive results, a measure of effect size (e.g., Pearson's r, Cohen's d)
- Report exact p-values wherever possible alongside the summary statistics and 95% confidence intervals. These should be reported for all key questions and not only when the p-value is less than 0.05.

Please outline where this information can be found within the submission (e.g., sections or figure legends), or explain why this information doesn’t apply to your submission:

All details on the statistical analysis are provided in Supplementary File 1. A summary of statistical analysis procedures is given in the Methods section. Confidence interval was 95% throughout.

Genetic analysis quantitative data are given as box-plots, which provide the median and the distribution of samples. For all of our graphs, the box represents 50% of the data with 25% at either side of the median, and the whiskers the 25% lowest and 25% highest values.

qRT-PCR data are given in bar charts, representing the mean fold change relative to a fixed control which was set to 1. The error bars indicate standard deviation.

Tests and P values can be found in the Supplementary File 1 as well as in the figure legends.

(For large datasets, or papers with a very large number of statistical tests, you may upload a single table file with tests, Ns, etc., with reference to sections in the manuscript.)

**Group allocation**

- Indicate how samples were allocated into experimental groups (in the case of clinical studies, please specify allocation to treatment method); if randomization was used, please also state if restricted randomization was applied
- Indicate if masking was used during group allocation, data collection and/or data analysis

Please outline where this information can be found within the submission (e.g., sections or figure legends), or explain why this information doesn’t apply to your submission:

Samples were grouped according to genotype.

Genotypes for each of the experiments are given in full in Supplementary File 1; and are given in abbreviated form within the text and figures.

The symbol > means GAL4 x UAS.

**Additional data files (“source data”)**

- We encourage you to upload relevant additional data files, such as numerical data that are represented as a graph in a figure, or as a summary table
- Where provided, these should be in the most useful format, and they can be uploaded as “Source data” files linked to a main figure or table
- Include model definition files including the full list of parameters used
- Include code used for data analysis (e.g., R, MatLab)
- Avoid stating that data files are “available upon request”

Please indicate the figures or tables for which source data files have been provided:

No raw data are provided, but all relevant details such as genotypes, sample sizes, repeats and statistical analysis details are provided in the manuscript, figures and Supplementary File 1.
